# Reconfigurable Acrylic-tape Hybrid Microfluidics

**DOI:** 10.1038/s41598-019-41208-y

**Published:** 2019-03-18

**Authors:** Yundong Ren, Subhrodeep Ray, Yuxiang Liu

**Affiliations:** Department of Mechanical Engineering, Worcester Polytechnic Institute, Worcester, MA 01609 USA

## Abstract

There is a great interest in low-cost, versatile microfluidic platforms of which the fabrication processes are rapid, straightforward, and translatable to industrial mass productions. In addition, it is beneficial for microfluidic devices to be reconfigurable in the field, so that multiple functions can be realized by a minimum number of devices. Here, we present a versatile acrylic-tape platform which allows highly accessible rapid prototyping of microfluidic devices, as well as device reconfiguration to realize different functions. The clean-room-free fabrication and sealing process only requires a laser cutter, acrylic, and tapes and can be done by an untrained person in the field. We experimentally characterized the relationship between the capillary flow speed and the channel height, the latter of which can be well controlled by the fabrication process. Reconfiguration of microfluidic functions was demonstrated on a single acrylic-tape device, thanks to the reversible sealing enabled by functional tapes. Different pumping mechanisms, including on-chip pumps for better portability and syringe pumps for precise fluid control, have been employed for the demonstration of two-phase flow and droplet generation, respectively. The low-cost and versatile acrylic-tape microfluidic devices are promising tools for applications in a wide range of fields, especially for point-of-care biomedical and clinical applications.

## Introduction

Since the first demonstration^1^, polydimethylsiloxane (PDMS) has been a dominant microfluidic platform with applications across a wide range of fields, including biology^2–4^, chemistry^2,5^, soft electronics^6^, and biomedical analyses^7^. However, PDMS based microfluidic devices have a number of limitations. The fabrication process is based on photolithography and soft lithography, which are time-consuming, need relatively expensive equipment such as plasma cleaners, and typically require a clean room environment. It can be difficult to translate the process into industrial mass productions^8^. In addition, most of the O_2_ plasma enabled PDMS-glass bonding is irreversible, while reversible sealing is beneficial because it allows reconfiguration of device functions and thorough sterilization of microfluidic channels.

Various alternative microfluidic platforms have been proposed to address the challenges of PDMS platform, including paper microfluidics^9,10^, 3D-printed microfluidics^11,12^, injection-molded blocks microfluidics^13^, and acrylic microfluidic^14–19^. The fabrication of these platforms does not need clean room facilities or plasma bonding as PDMS devices do. Paper microfluidics have benefits of low cost, lightweight, and disposable, compared with the PDMS platform. However, paper microfluidic devices are mechanically weak and have poor flow control^12^. 3D-printed and injection-molded blocks microfluidics have benefits of function reconfigurability, which means the functions of the microfluidic devices can be changed without the need of fabricating new parts. This is realized by their modular designs^11–13^, which enable different blocks to be rearranged in the field to provide different functions. However, both their fabrication and assembling processes are relatively complex and cannot be done by a novice without proper training. Acrylic microfluidic devices have benefits of mechanical durability and low cost. Furthermore, acrylic, also referred to as poly(methyl methacrylate) (PMMA), is non-porous and hence can prevent adsorption or absorption related contaminations. In most of the previously demonstrated acrylic microfluidics, the bonding between the channels and the covers was realized by hot laminating machine^15^, thermal fusion bonding^16^ or solvent-assisted lamination^15^, all of which are permanent and needs special equipment. In addition, these bonding processes take longer than 20 minutes. Recently, the bonding between two pieces of acrylic has been realized more quickly by double-sided tapes^17^. However, this bonding mechanism is not reconfigurable because the channels were formed inside the tapes. Reconfigurable microfluidic devices made from low cost and mechanically durable materials in a rapid fashion are yet to be demonstrated.

In this paper, we developed an acrylic-tape hybrid microfluidic platform, where devices are fabricated by a readily accessible, straightforward process and sealed by a reversible tape-based mechanism. The tape-based sealing of acrylic microfluidic devices is reliable for long-term applications while allowing in-the-field reconfiguration of the device functions by changing tapes. We investigated the channel geometries and flow speeds to characterize the laser ablation fabrication method. The flow control enabled by the functional tapes was demonstrated to showcase the potential of function reconfigurability and multiplexing. Both on-chip pumps and syringe pumps are used for flow actuation, for better portability and accurate flow control, respectively. In addition, we demonstrated microfluidic functions such as two-phase flow and droplet generation on the acrylic-tape platform. These results indicate that acrylic-tape microfluidics is a suitable platform for applications including device prototyping, point-of-care testing, and clinical bioanalyses.

## Fabrication Methods And Materials

**Figure 1:**
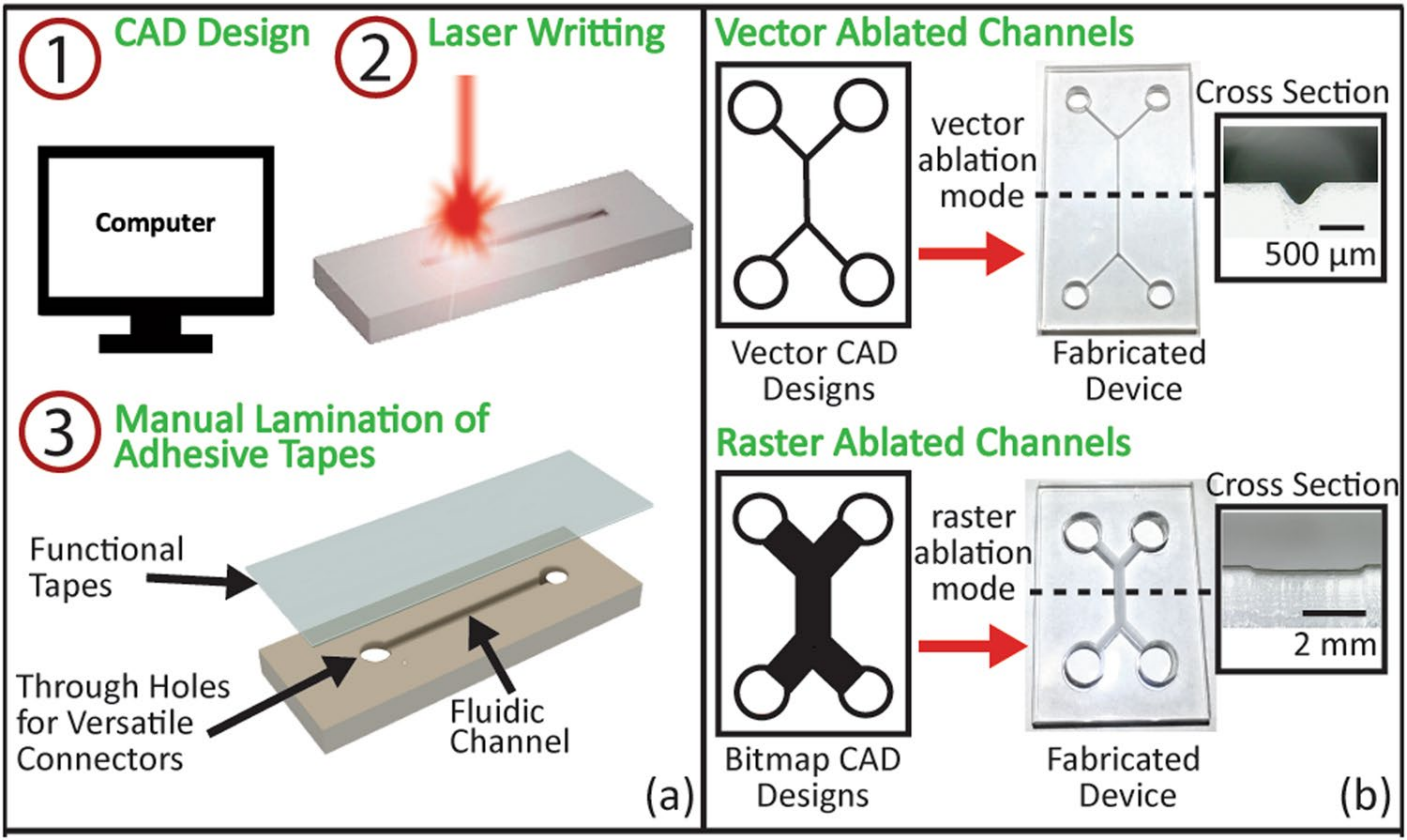
(**a**) Schematics of the fabrication process of the acrylic-tape hybrid microfluidic devices. (**b**) Fabricated devices by two different laser ablation modes: vector ablation (top) and raster ablation (bottom). The left column shows the CAD channel designs in the vector format (top) and the bitmap format (bottom). The right column shows the corresponding photos of the fabricated device and microscopic images of the channel cross-section. It is noted that the vector ablated channel has a triangular cross-section (top) while the raster ablated channel has a rectangular cross-section (bottom).

Fabrication steps of the acrylic-tape hybrid microfluidic devices are shown in Fig. 1(a). Firstly, the computer-aided-design (CAD) drawings of the devices, including flow channels and inlets/outlets, are developed on a computer. Secondly, a CO_2_ laser cutter is used to fabricate the devices on commercially available acrylic boards. Inlets/outlets are fabricated by cutting through the boards, while channels are engraved into the surface by laser ablation. Details on the laser ablation method can be found in previously published works^15,18^. Briefly, when a laser spot that has an appropriate power moves across the acrylic surface, instead of cutting through the acrylic board, it heats and vaporizes a small volume of acrylic material. The resulted cavities left on the acrylic surface serve as the microfluidic channels. Finally, the channels are sealed with functionalized single-sided tapes, completing the fabrication process. A detailed discussion of the functional tapes is presented in the following sections.

Multiple devices can be fabricated in a single laser cutting process on one 12″ × 24″ acrylic board. The fabrication of 10 devices takes less than 30 minutes, and the results are highly reproducible. This straightforward three-step fabrication process allows rapid, controllable, and repeatable prototyping, which can greatly increase the efficiency of the iterative design process. It is noted that this fabrication method is based on laser ablation and limited to materials that can be ablated by lasers. Materials that are transparent at the CO_2_ laser wavelength (around 10 μm) such as glass cannot be used.

There are two ablation modes in the laser cutter, namely the vector mode and the raster mode. The ablation modes are controlled by the formats of the CAD designs uploaded to the laser cutter as shown in Fig. 1(b). Each ablation mode has its own advantages and disadvantages, which will be detailed in the next paragraph. For the vector ablation mode, CAD designs are uploaded in the vector image format. In this mode, the laser cutter treats the CAD designs as continuous vector lines and continuously ablates these lines to form channels. For raster ablation mode, CAD designs are uploaded in the bitmap image format. In this mode, the laser cutter treats the CAD designs as discrete pixels and ablates all the pixel points with discrete laser pulses to form channels. Typical cross-sections of the channels fabricated by these two modes were shown in Fig. 1(b).

The roughness of the channels is determined by the laser cutter settings for both vector and raster ablation modes, while typically the vector mode ablated channels have smoother surfaces. As revealed by a recent study^14^, the roughness of the laser-cut channels ranges from 4 nm to 10 μm. A typical roughness of the vector mode ablated channels is 9.5 μm, as measured by a confocal microscope (LEXT OLS4000, Olympus). Although the roughness is not as good as that of the PDMS, the laser-ablated acrylic channels meet the needs of a wide range of applications, as proved by all the experimental results below in this paper. The widths of the channels fabricated by the vector and raster ablation modes are different. Vector mode ablated channel widths are determined by the laser spot diameter. In our case, the typical width of the channel opening is around 200 μm. By comparison, the raster mode ablated channels have a minimum width of around 450 μm, but the width can be much wider and not limited by the laser spot diameter. The two types of channels are preferred in different applications. Vector ablated channels are used when smooth, small channels are preferred, such as in droplet generation applications. The raster ablated channels with wider cross-sections are preferred in point-of-care testing applications, where the wider cross-section channels make reading out the test results easier. We would like to note that strong optical scattering is caused by the relatively high roughness of the laser ablated channel surfaces. As a result, the transmission microscope images of these channels have limited resolution. However, the single-sided sealing tapes are optically clear and have a thickness of 50~150 μm, allowing for reflection microscope images with good quality to be readily taken from the tape side. All the experimental photos of the results below were taken from the tape side at the reflection imaging mode, if not specified otherwise.

The cost for fabricating acrylic microfluidic chips is low. For example, a 12″ × 24″ blank acrylic board costs less than $10, and a roll of single-sided tape costs around $2. From these materials, hundreds of acrylic microfluidic devices can be made even in the field, which leads to a material cost of less than $0.1 per device. In addition, after the tapes are peeled off, the acrylic substrate with channels could be thoroughly cleaned and sterilized for re-use, which further reduces the device cost, as well as the shipping and storage costs. The laser cutter is the most expensive equipment required for the fabrication. In this work, we used a professional laser cutter (VLS-4.60, Universal Laser Systems) simply because it is available. However, all the results should be repeatable by a regular laser cutter available on the market. It has been demonstrated that even a modified CD optical pickup head system can ablate micro-scale channels on acrylic boards^20^. In addition to the unique capabilities of the acrylic-tape devices, the decreasing cost and increasing accessibility of laser cutters in academia and industry make the platform even more appealing.

## Results and Discussions

### Flow characterization of the vector ablated acrylic channels

One important parameter of a microfluidic device is its characteristic length, which is directly related to the Reynolds number and the capillary action of the channel^21^. In our acrylic microfluidic devices, the Reynolds number is low (0.1–0.7) and the flow is laminar. Thus, a linear relationship is expected between the flow speed of capillary flow and the channel’s characteristic length. However, as discussed above in Section 2, our laser ablated acrylic channels have rougher surfaces than that of the conventional PDMS microfluidics, and this roughness could cause the flow behavior to differ from the expectation. As a result, it is important to experimentally determine the dependence of the flow on the characteristic length in the acrylic microfluidic channels.

**Figure 2:**
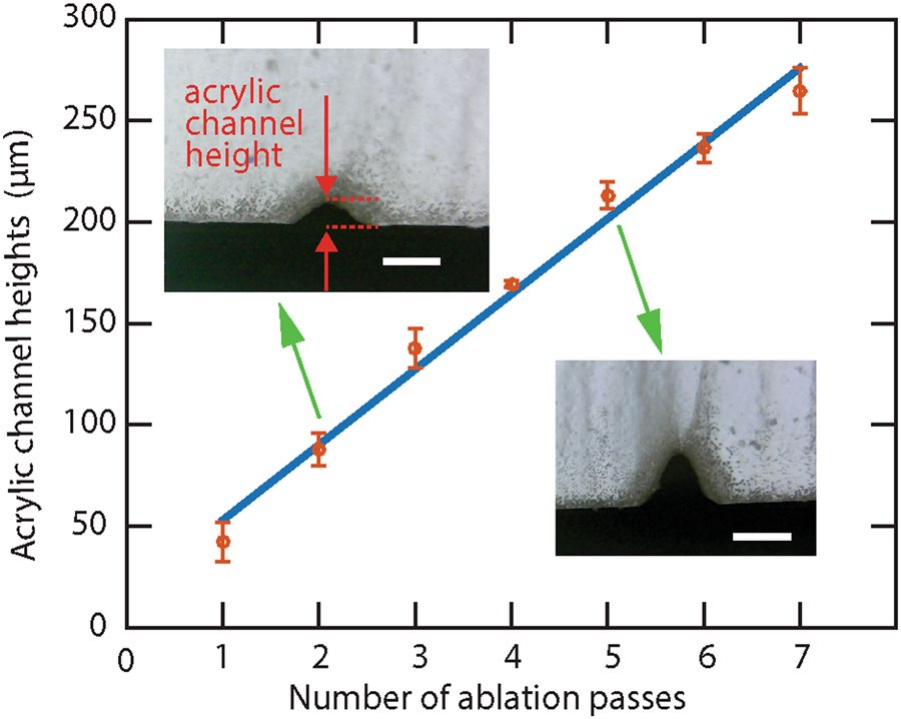
Measured heights of acrylic channels fabricated by different numbers of ablation passes. The red dots represent heights of channels fabricated by different numbers of ablation passes. The heights were measured from the microscope images of the channel cross-sections. (Insets) Two typical microscope images. The scale bars in both images are 200 μm. The blue linear regression line shows a linear relationship between the channel height and the number of ablation passes. The uncertainties are determined by the standard deviation obtained from 5 independent measurements on channels fabricated with the same number of ablation passes.

For this purpose, we vector ablated acrylic channels with different heights, which resulted in different characteristic lengths. With a laser cutter, the height can be changed by using a different laser power with a fixed number of ablation passes or a different number of ablation passes with a fixed laser power. In both cases, the channel width on the top surface remains the same when the height is changed, because we focused the laser spot on the top acrylic surface before ablating the channels. In our experiment, we always changed the number of ablation passes with a fixed power to change the channel heights, as shown in Fig. 2.

**Figure 3:**
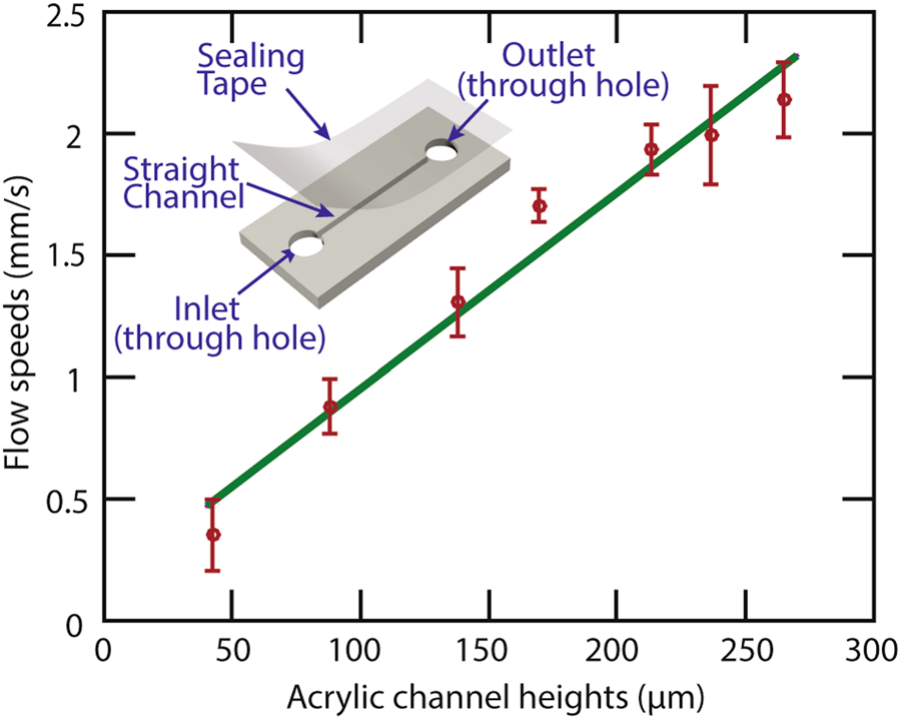
Flow speed characterization. The red dots represent the experimentally acquired flow speeds in channels with different heights. The green line is the linear fitting between the flow speed and the acrylic channel height. The inset schematic shows the acrylic device used for the characterization. The uncertainty of each data point was determined by the standard deviation obtained from 5 independent experiments with the same channel.

In the characterization experiment, we plasma-treated the acrylic channels to create hydrophilic acrylic surfaces and then sealed it with hydrophilic tapes (ARflow 93049, Adhesives Research). After applying the liquid to the inlet without an additional pressure, the capillary-force-driven flow was recorded by 1080p, 60 frame-per-second videos. We measured the time for the capillary-force-driven flow to pass through the channel and calculated the flow speed by the ratio of the channel length to the time. From the experiments, we found that the flow speed increased linearly with the channel height, as shown in Fig. 3. This confirms the linear relationship between the flow speed and the channel’s characteristic length, in this case, the channel height. This linear relationship in the experiment agrees with the capillary action and Hagen-Poiseuille theory^22^. Briefly, the capillary force (*F*_*C*_) on the fluid inside the channel act as an equivalent hydro-pressure Δ*P* = *F*_*c*_/*A* that drives the fluid flow. The pressure scales with channel height (*H*) as as Δ*P*~*H*^−1^. As a result, the flow speed (*v*) can be calculated by *v* = *Q/A* and *Q* = Δ*P/R*, where *Q* is the volumetric flow rate, *A* is the cross-section area that scales with *H*^2^, and *R* is the hydraulic resistance that scales with *H*^−4^. Therefore, the flow speed *v* scales linearly with *H* as *v*~H. This experimental result confirms that the roughness of the laser ablated channel do not alter the flow behaviors that are expected from the theory. Such capillary action based microfluidic devices are desired in point-of-care applications for its active pump free, passively driven fluid flows.

### Reconfigurable acrylic-tape hybrid microfluidic devices with multiplex functions

It is beneficial to reconfigure the functions of a microfluidic device without re-patterning the fluidic channels. This allows multiplexing of different functions on one single acrylic device with fixed channel patterns. These devices are appealing particularly for point-of-care applications, where the number of devices to realize multiple functions can be significantly reduced, saving cost on transportation and storage. However, most of the existing microfluidic devices are not reconfigurable. For example, most of the PDMS based devices that are plasma bonded can’t be dissembled for reconfigurations. In paper microfluidics, functional chemicals or bio-markers^23^ are permanently coated onto the paper flow channels, making it difficult to reconfigure. By comparison, our acrylic-tape hybrid microfluidic platform can be reconfigured by changing the functional tapes, which can be done by an untrained person in the field, without requiring expensive equipment. The tape can be peeled off with minimum efforts for reconfigurations, and a new functional tape can be applied to the cleaned acrylic substrate, bestowing reconfigured, different functions on the same device. To showcase this capability, we demonstrate here reconfigurable, controlled flow actuation on an acrylic-tape microfluidic device.

**Figure 4:**
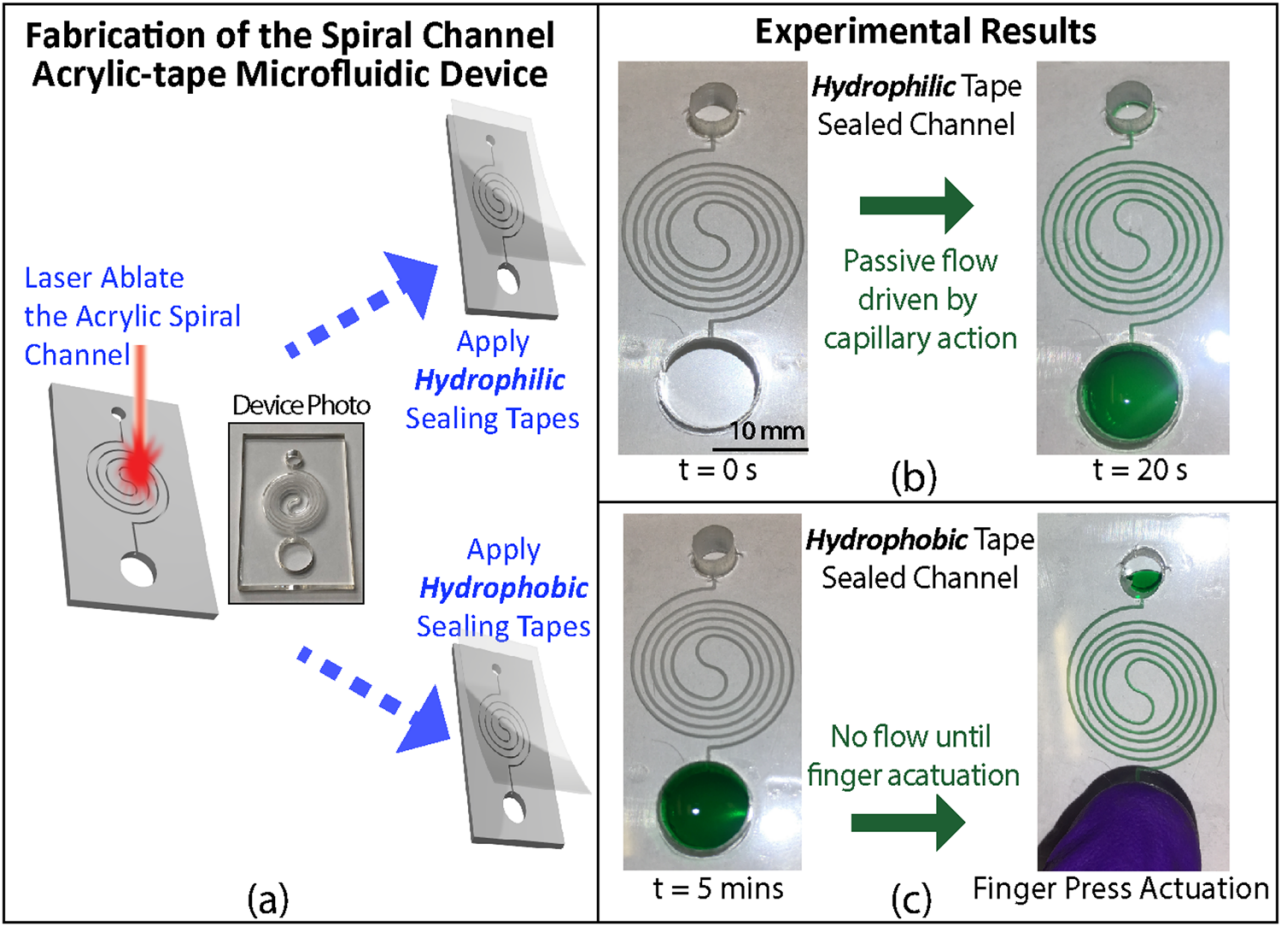
(**a**) Fabrication and assembly of the spiral channel acrylic microfluidic devices with reconfigurable flow actuation mechanisms. A spiral microfluidic channel is laser ablated onto an acrylic board. Either a hydrophilic or a hydrophobic tape is applied to seal the channel. (**b**) Automatic pump-free flow actuation with the spiral acrylic microfluidic channel sealed by a hydrophilic tape. Water with green dye was dropped into the inlet at t = 0. The whole channel was automatically filled due to the passive capillary action at t = 20 s. (**c**) Finger-press flow actuation with the same spiral channel sealed by a hydrophobic tape. Water with green dye was dropped into the inlet at t = 0 and could not flow into the channel after 5 minutes because of the tape hydrophobicity, until actuated by a finger press on the inlet. The scale bar is 10 mm. The results in this figure were obtained with acrylic channels fabricated using the vector ablation method shown in Fig. 1 and can be repeated with channels made by raster ablation (data not shown).

Controlled flow actuation, especially delayed flow for sequential fluids delivery, is important in many applications^24–27^. We demonstrated different flow actuation mechanisms on a spiral microfluidic channel. As shown in Fig. 4, in an acrylic channel sealed by a hydrophilic tape, once the green-dyed water was dropped into the inlet reservoir, it automatically wicked into the channel by capillary action. We peeled off the hydrophilic tape, cleaned the acrylic channels by rinsing them with isopropanol alcohol (IPA), and applied a hydrophobic tape on the channels to reconfigure the device. The green-dyed water applied to the inlet was confined in the inlet reservoir until a finger-press actuation. The results prove that we can switch the flow actuation mechanisms between passive capillary force driving and active finger actuation by changing the tapes.

**Figure 5:**
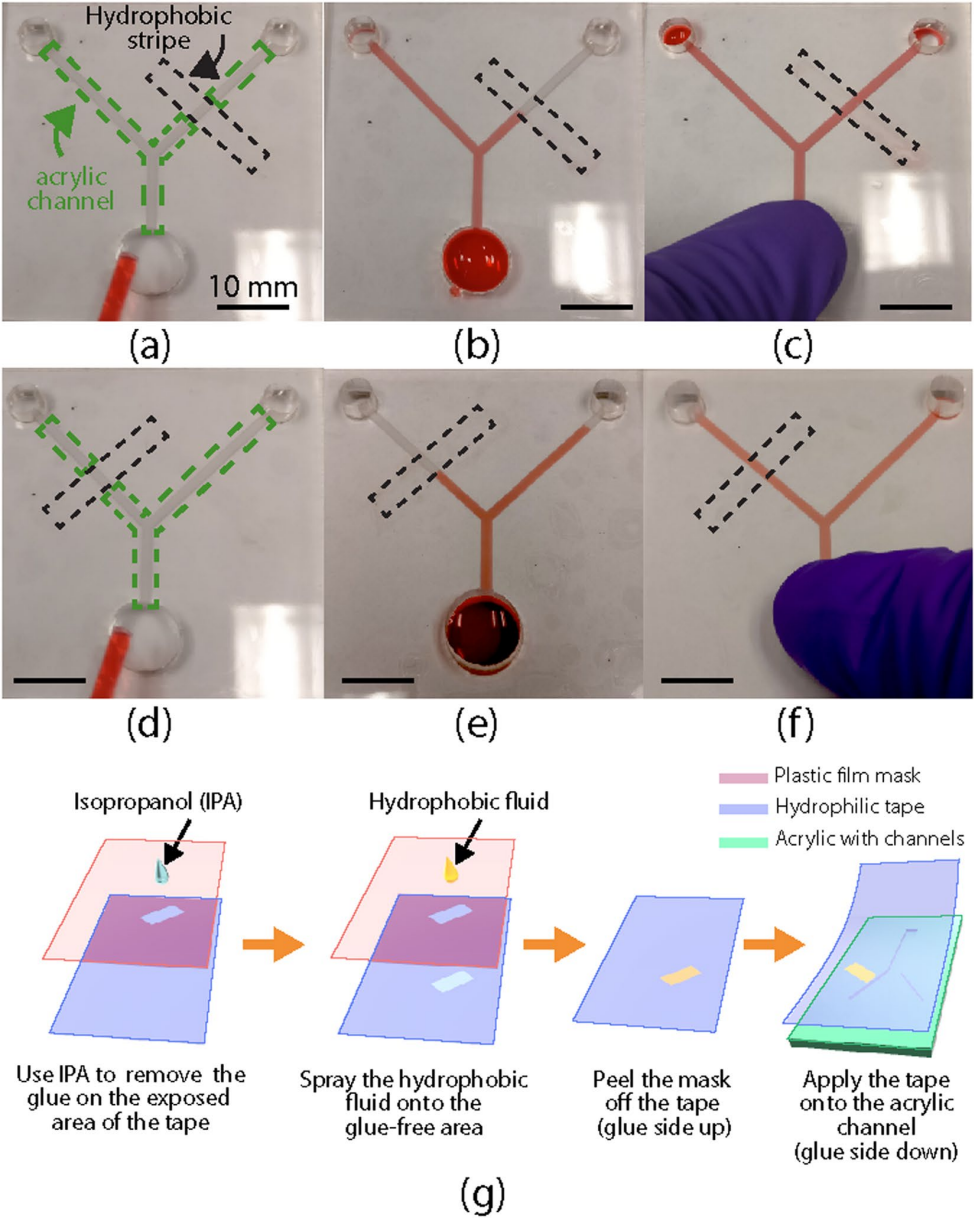
Reconfigurable acrylic microfluidics by functional tapes. (**a–c**) Flow patterns in a Y-shaped channel (the green dotted box) sealed by a hydrophilic tape with a hydrophobic stripe (black dotted box) on the top right channel. (**d–f**) Reconfigured flow patterns in the same Y-shaped channel sealed by a hydrophilic tape with a different hydrophobic stripe (the black dotted box) location. The flow patterns (**a**,**d**) before and (**b**,**e**) after the water was applied, and (**c**,**f**) after a finger press on the inlet (on the bottom of the Y channel). The fluid was water with red dye. All scale bars are 10 mm. (**g**) Schematics of the fabrication of a hydrophilic tape with a hydrophobic strip, as well as the application of the tape to an acrylic substrate with a Y-shaped channel. The Y-shaped channel was fabricated using the raster ablation method shown in Fig. 1.

In addition to the flow actuation mechanism, we demonstrated reconfigurable flow pattern on a single acrylic microfluidic device. Specifically, as shown in Fig. 5(a–f), we used hydrophilic tapes with different hydrophobic patterns to realize different flow patterns in the same Y-shaped acrylic channel. When the hydrophobic stripe is on the top right channel (Fig. 5(b)), after the red dyed water was dropped into the inlet at the bottom of the Y-channel, water was immediately driven by the capillary force and automatically filled the top left channel. However, the water was stopped by the right channel’s hydrophobic stripe, leaving the right channel dry. When we used a different tape with a hydrophobic strip located at the top left channel, the flow pattern was reversed, as shown in Fig. 5(e). In both cases, all channels were filled right after a finger press was applied onto the inlet. The deformation of the tape resulting from the finger press generated additional hydraulic pressure that pushed the water through the hydrophobic stripes, as shown in Fig. 5(c,f). These results prove that we can reconfigure the flow patterns in the field, without fabricating a new device, making it potential to multiplex various functions on a single acrylic device.

In the abovementioned experiment, hydrophobic patterns can be created by selectively replacing hydrophilic glue with a hydrophobic coating on desired areas of the tape. The detailed experiment steps are explained below and shown in Fig. 5(g). First, a plastic film was laser cut to expose the desired patterns of the final hydrophobic areas. The cut film is applied on the sticky side of the hydrophilic tape, serving as a mask. The exposed hydrophilic glue on the tape was removed by rubbing a cotton swab soaked with IPA on the surface. Commercially available hydrophobic fluid (GRF135, Granger) was sprayed onto the masked tape to make the exposed area hydrophobic. With the plastic film mask removed, the hydrophilic tape can be applied to seal the acrylic channels with the hydrophobic patterns at the desired locations.

We note that acrylic-tape microfludics can be sterilized, especially before the devices are reconfigured. Commercially available pre-sterilized tapes (ARcare 8311, Adhesive Research) can be used. By removing the tape, channels in the used acrylic microfluidic devices are fully exposed and can be thoroughly cleaned or sterilized by solvents. We immersed the acrylic substrates with laser ablated channels in IPA and ethanol over 48 hours, and there was no crack observed, proving the solvent cleaning is safe to sterilize our acrylic substrates. We also note that the application of reconfigurable functional tapes is not limited to flow pattern controls. Other functional materials can be patterned onto the tapes for interdisciplinary process control, such as the chemical catalyst for multi-step reactions and biomarkers for immunoassays. Furthermore, the tape coating process is suitable for mass production in industry, which is important for translating laboratory research to real-world applications.

### Acrylic-tape microfluidic devices with on-chip pumps

Control of continuous flow in microfluidics is important for both laboratory experiments and in-the-field testing. For example, in microfluidic H-filter applications^28^, a controlled continuous flow is required for maintaining the two-phase flow boundary and transportation of the filtered substances. While conventional syringe pump systems can provide more accurate flow control, their relatively large sizes and the requirement of an AC supply can compromise their usability in the field. Especially when only moderate flow control is required, microfluidic devices with on-chip pumps are preferred. In this section, we demonstrate two on-chip pump designs that are compatible with the acrylic-tape hybrid microfluidic devices. In both demonstrations, the acrylic channels were fabricated using the raster ablation method, which is shown in Fig. 1 and detailed in Section 2.

#### On-chip paper pump

**Figure 6:**
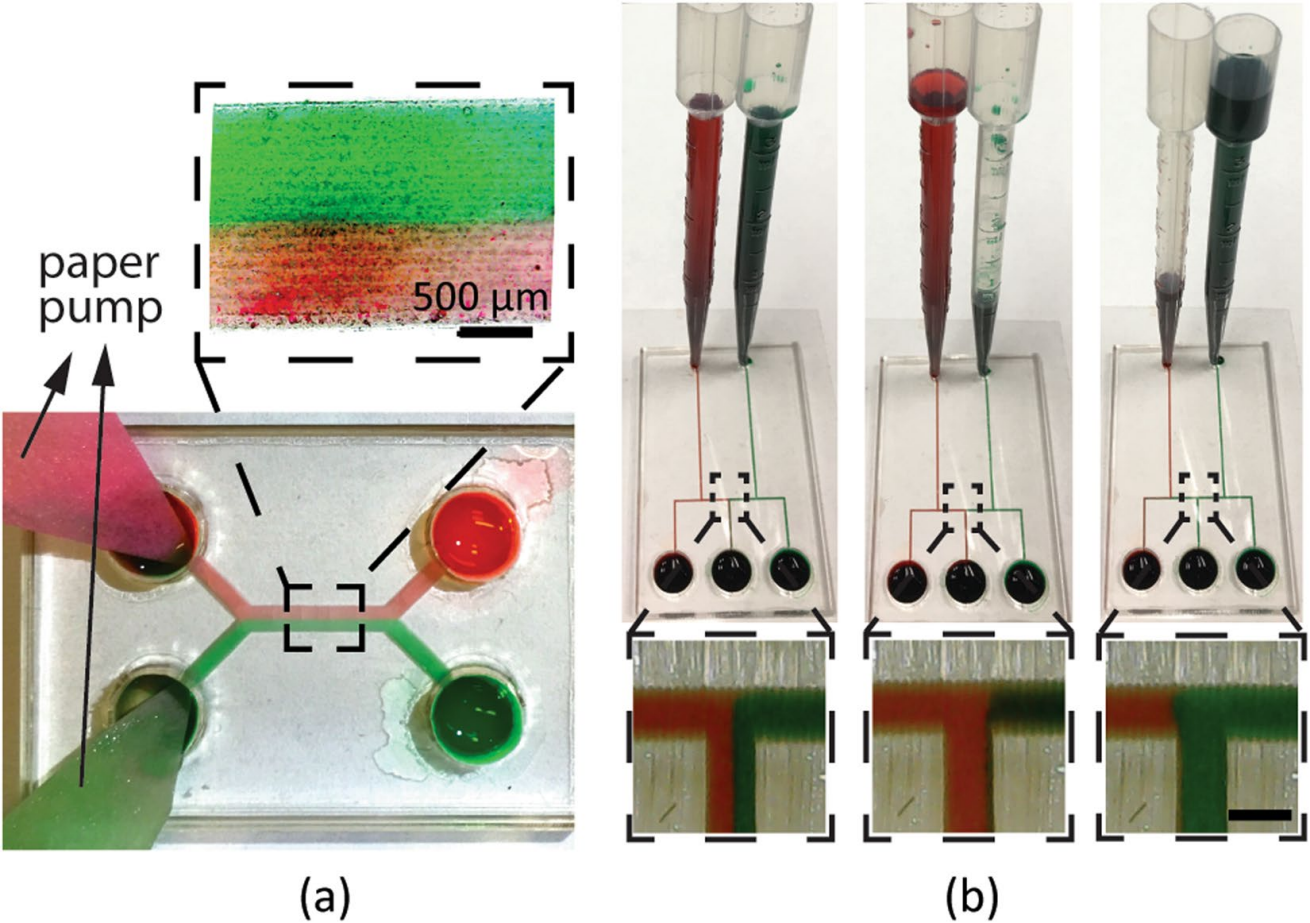
Experiment results of (**a**) on-chip paper pumps and (**b**) on-chip pipette tube pumps. The channel in (**a**) has a width of around 2 mm, and a height of around 200 μm. The inset in (**a**) shows a microscope image of the two-phase laminar flow in the channel. The channels in (**b**) have a width of around 500 μm, and a height of around 200 μm. The insets in (**b**) show the microscope images of the middle channel, where the output fluid was controlled in real time by the pumps. Scale bars in all the insets are 500 μm.

Paper is lightweight and low-cost, and their porous structures have excellent wicking properties to provide continuous flow for a relatively long time (~60 s). We applied paper pumps, which was demonstrated previously in PDMS microfluidics^29^, in our acrylic-tape devices. We inserted two pieces of Whatman filter paper into the two outlets of an acrylic H-channel, respectively, as shown in Fig. 6(a). One of the inlets was filled with red-dyed deionized water (DI water), and the other inlet was filled with green-dyed DI water. The fluid was first driven by the capillary force to fill the channel and wicked into the papers at the outlets, until the paper was fully soaked with the fluid. The hydraulic pressure provided by the wicking paper was controlled by the paper’s cross-section area. Two paper pumps with the same cross-sections provided equal hydraulic pressures at the two outlets of the H-channel. Thanks to this constant and continuous driving pressure, the two-phase flow maintained a stable boundary in between, as can be clearly seen in the inset of Fig. 6(a). Besides providing constant hydraulic pressure, paper pumps with carefully designed cross-sections can also provide various functions, as demonstrated in previous work^27^.

#### On-chip pipette tube pump

For applications that require flow durations longer than one minute, we used on-chip pipette tube pumps to drive the flow in acrylic microfluidic devices. The diameters of the rigid acrylic inlets of the microfluidic channels are designed to be slightly smaller than the outer diameters of the deformable pipette tips. As a result, the interference fit ensured fluid-tight sealing between the pipette tips and the acrylic inlets. The fluid height in a pipette was around 15 cm with a fluid volume of around 5 ml. A continuous flow in the channels was realized by the hydrostatic pressure at the inlet for more than 30 minutes. The hydraulic pressure can be easily adjusted on the fly by controlling the fluid height in each pipette.

Figure 6(b) shows the experimental results of a microfluidic chip with two on-chip pipette tube pumps. The output fluid of the middle channel can be switched in real time by controlling the input pressure from the pipette pump. At first, the red and green dyed water was kept at the same heights in the pipettes to realize a balanced hydraulic pressure input. As a result, the red-green water interface is located at the center of the middle output channel, resulting in the same volume ratio of red and green dyed water in the middle outlet, as shown in the left column of Fig. 6(b). When green-dyed water was drawn out from the pipette to lower its hydraulic pressure, the red-green water interface shifted to the right of the middle channel, as shown in the middle column of Fig. 6(b). As a result, only red fluid was output from the middle channel. Similarly, when the green pipette has a higher water level, the red-green interface shifted to the left of the middle channel, resulting in only green fluid output from the middle outlet, as shown in the right column of Fig. 6(b). A complete switch from red to green water at the middle outlet required about 1~2 seconds.

### Syringe pump driven monodisperse droplet generation

In addition to on-chip pumps that are preferred for point-of-care applications, traditional syringe pump systems are also readily compatible with the acrylic-tape hybrid devices. Syringe pumps are preferred for applications where well controlled flow speeds are important while the portability is not a concern.

**Figure 7:**
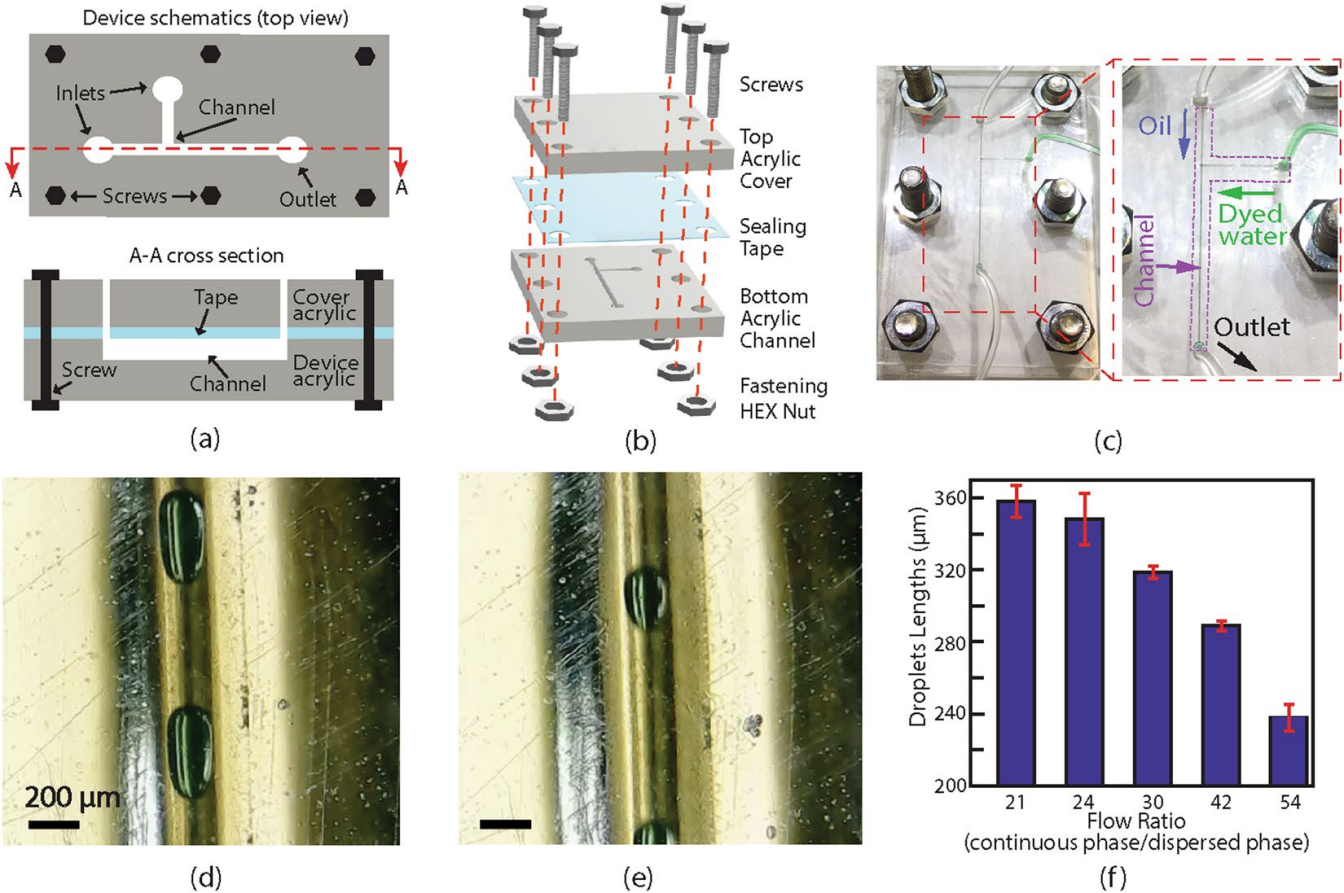
(**a**) 2D schematics of a T-channel acrylic-tape microfluidic device for monodisperse droplet generation. The channels were fabricated using the vector ablation method shown in Fig. 1. (**b**) An exploded-view showing the assembly of the device. (**c**) Photos of an assembled acrylic-tape microfluidic device. The arrows in the zoomed-in (right) photo show flow directions from the inlets and outlet inside the T-shaped channel, while the channel is highlighted by a purple dotted box. (**d**) and (**e**) are microscopic images showing two typical monodisperse droplets generated at volumetric flow ratios (continuous phase flow over dispersed phase flow) of 30 and 54, respectively. The scale bars are 200 μm. (**f**) Experimentally measured droplet lengths at different ratios of continuous to dispersed phase flow speeds. The uncertainty of each data point is determined by the standard deviation obtained from 5 independent measurements on the droplets generated with the same flow ratio.

Monodisperse droplet generation is an important application in microfluidics. It requires a continuous phase flow with a relatively high speed to squeeze and break off the dispersed phase fluid into droplets. To generate droplets with consistent geometries, a well controlled and maintained flow ratio between the dispersed phase and the continuous phase is required. Here, we demonstrated droplet generation on an acrylic-tape hybrid microfluidic device and characterized the dependence of the droplet size on the flow rate. The channel design, assembly schematic, and the photos of an assembled device are shown in Fig. 7(a–c), respectively. A T-junction microfluidic channel design was chosen for droplet generation. To ensure a high flow rate without a concern of tape leakage, the tape sealed channel layer was sandwiched by two acrylic boards which are clamped with screws.

Microscope images of the generated droplets with different diameters are shown in Fig. 7(d,e). The fluid in the droplets was green-dyed deionized water, the continuous phase medium was vacuum pump oil (Vacuum Pump Oil No. 19, VWR). Droplets with different lengths were consistently generated by a fixed disperse phase flow rate of 1.667 μl/min and various continuous phase flow rates from 35 to 90 μl/min. Droplet generation at a frequency of 7 Hz was demonstrated with a coefficient of variation (CV) of the droplet length as low as 4%. The CV is defined as the standard variation of the generated droplet lengths normalized by the average length. The characterization results of droplet lengths at different flow rates are shown in bar charts in Fig. 7(f), and the droplet widths were always the same with the channel width.

In addition to the droplet generation, the functional tape sealed acrylic-tape devices can potentially enable droplet manipulation if proper tapes are used. For example, electric-field-based droplet manipulation^30^ requires materials that can withstand high voltages, and the electrical tapes (Super 88 Vinyl Electrical Tape, 3 M) with 10 kV breakdown voltages are suitable for these applications. In magnetic-field-based droplet manipulation^31^, magnetic tapes (Flexible Magnet Tape, 3 M) can be used to support magnetic fields in the fluid channels. Furthermore, all the above mentioned special tapes are commercially available, which is beneficial to decrease the cost and to increase the accessibility of the acrylic-tape microfluidic devices.

## Conclusions

In conclusion, we developed a low-cost and versatile acrylic-tape hybrid microfluidic platform. Different from that of PDMS microfluidics, the fabrication process is rapid, straightforward, and does not require clean-room access or plasma bonding, which makes the acrylic-tape microfluidics readily translatable to industrial mass production. The tape-based reversible sealing mechanism is reliable for long-term applications and allows reconfiguration of device functions to be carried out in the field. We experimentally characterized the dependence of the capillary flow speed on channel geometric parameters. We demonstrated reconfigurable functions by changing functional tapes on the same microfluidic device, thanks to the reversible sealing and multiple flow mechanisms enabled by different tapes. This result indicates the potential of function multiplexing on a single acrylic-tape microfluidic device and the ability of changing the device functions in the field. Different pumping mechanisms have been investigated for the acrylic-tape platform. To reduce device footprints for point-of-care applications, two types of on-chip pumps, namely paper pumps and pipette tube pumps, have been used to demonstrate reliable and adjustable two-phase flow. In addition, repeatable and controllable droplet generation was demonstrated with syringe pumps. The ability to reconfigure and multiplex functions on a single device, augmented by its immediate compatibility with both on-chip pumps and syringe pumps, makes the acrylic-tape hybrid microfluidics a versatile platform with great potential for in-the-field applications such as biomedical testing and point-of-care diagnosis. Our future work will be focused on the integration of multiple functions across disciplines, including surface chemistry, electrochemistry, biology, and optics, by leveraging the adaptive and versatile nature of the acrylic-tape platform.

## Methods

### Laser ablation of microfluidic channels

The CAD drawings of the microfluidic channels were prepared using the AutoCAD software. The laser cutter used in the fabrication was a VLS-4.60 by Universal Laser Systems. The linewidth in the prepared CAD drawings was set to be 0.001 inches. According to the specifications of the laser cutter, the different ablation modes were determined by the color of the lines in the CAD drawings, with red lines corresponding to the vector mode ablation and black lines to the raster mode ablation.

### Plasma treatment of the acrylic channel

The plasma treatment was done in a commercially available plasma cleaner, PDC-32G from Harrick Plasma. Before the plasma treatment, the acrylic channels were thoroughly rinsed by isopropyl alcohol. The plasma chamber was vacuumed to a pressure of around 800 mTorr and the plasma was generated with air.

### Tape sealing of the acrylic channels

To seal the channels, a single-sided tape was manually applied onto the channel-side acrylic surface and was gently scrubbed with the edge of a ruler to ensure a good contact. When hydrophilic channels were desired, tapes with hydrophilic surfaces (ARflow 93049, Adhesives Research) were used. When hydrophobic channels were desired, tapes with hydrophobic surfaces (EL-92734, Adhesives Research) were used. Otherwise, if the hydrophobicity or the hydrophilicity were not of concern, any common transparent single-sided tapes can be used (e.g., 3 M Scotch Clear Tape). For the special sealing tapes prepared for the reconfigurable microfluidics, detailed fabrication steps can be found in Section 3.2.

### On-chip paper pump

The on-chip paper pumps were fabricated with filter paper (Cat. No. 1001–055, Whatman). The original paper has a circular shape with a diameter of 55 mm. The paper was cut into fan-shaped pieces so that they could be easily inserted into the channels.

### On-chip pipette pump

The commercially available pipette tubes (H&PC-49078, Fireboomoon) have a reservoir of 5 ml and a length around 15 cm. The small ends of the pipettes were cut off with scissors and inserted into the inlet of the microfluidic channels, to allow fluid injection.

### Dyed water and oil

Dyed water was prepared by dissolving common food dyes (Chefmaster) in de-ionized water. The oil used in the droplet experiment was vacuum pump oil (Vacuum Pump Oil No. 19, VWR). Similar results were obtained with other types of oil, such as cooking oil (data not shown).
